# Realistic morphology-preserving generative modelling of the brain

**DOI:** 10.1038/s42256-024-00864-0

**Published:** 2024-07-15

**Authors:** Petru-Daniel Tudosiu, Walter H. L. Pinaya, Pedro Ferreira Da Costa, Jessica Dafflon, Ashay Patel, Pedro Borges, Virginia Fernandez, Mark S. Graham, Robert J. Gray, Parashkev Nachev, Sebastien Ourselin, M. Jorge Cardoso

**Affiliations:** 1 Department of Biomedical Engineering, King’s College London, London, UK; 2 Institute of Psychiatry, King’s College London, London, UK; 3 Department of Psychological Sciences, Birkbeck, University of London, London, UK; 4 Data Science and Sharing Team, Functional Magnetic Resonance Imaging Facility, National Institute of Mental Health, Bethesda, MD USA; 5 Machine Learning Team, Functional Magnetic Resonance Imaging Facility, National Institute of Mental Health, Bethesda, MD USA; 6 Queen Square Institute of Neurology, University College London, London, UK

**Keywords:** Computer science, Translational research

## Abstract

Medical imaging research is often limited by data scarcity and availability. Governance, privacy concerns and the cost of acquisition all restrict access to medical imaging data, which, compounded by the data-hungry nature of deep learning algorithms, limits progress in the field of healthcare AI. Generative models have recently been used to synthesize photorealistic natural images, presenting a potential solution to the data scarcity problem. But are current generative models synthesizing morphologically correct samples? In this work we present a three-dimensional generative model of the human brain that is trained at the necessary scale to generate diverse, realistic-looking, high-resolution and morphologically preserving samples and conditioned on patient characteristics (for example, age and pathology). We show that the synthetic samples generated by the model preserve biological and disease phenotypes and are realistic enough to permit use downstream in well-established image analysis tools. While the proposed model has broad future applicability, such as anomaly detection and learning under limited data, its generative capabilities can be used to directly mitigate data scarcity, limited data availability and algorithmic fairness.

## Abstract (web summary)

Medical imaging research is limited by data availability. To address this challenge, Tudosiu and colleagues develop a 3D generative model of the human brain that can generate high-resolution morphologically correct brains conditioned on patient characteristics.

## Main

In computer vision, the rapid progress of deep learning methods was underpinned by huge datasets such as ImageNet^1^, TextOCR^2^ and COCO^3^, which contain around 1.2 million, 1 million and 170,000 samples respectively. Current volumetric medical imaging datasets pale in comparison: UK Biobank (UKB)^4,5^, one of the largest datasets available, has approximately 40,000 samples, the Alzheimer’s Disease Neuroimaging Initiative (ADNI)^6^ has roughly 3,000 images and Medical Segmentation Decathlon^7^ roughly 3,000 images across 10 organs. This limitation, combined with the 3D nature of medical images, results in datasets that do not cover all anatomical, pathological and signal variations. At present, state-of-the-art medical imaging algorithms are developed on highly curated datasets, leading to potential biases regarding demographics and acquisition parameters that may adversely affect particular populations. Owing to the restrictive nature and volume of medical imaging data, deep learning models are often limited in scale, further hindering the deployment of successful research in clinical environments. To mitigate the need for specialized and costly acquisition equipment, as well as the restrictions entailed by regulations and complex maintenance^8^, generative modelling can be seen as a viable solution. Not only might it allow the open sourcing of a large corpus of data, but it also enables the prevalence of confounding variables such as pathologies and ethnicities to be balanced.

Variational autoencoders (VAEs)^9^ are the classic baselines for generative modelling in computer vision, but they are known to suffer from blurry reconstructions. Generative adversarial networks (GANs) are the current state of the art in generative modelling of the brain^10–12^. The generator tries to learn the underlying distribution of images by generating realistic-looking images, aiming to fool the discriminator into labelling them as real images^13^. The main drawbacks of current approaches are the commonly known pitfalls of GANs, including unstable training regimes, mode collapses (always generating the same images), failure to converge^14^ and the lack of mechanisms for a fine-grained conditioned generation. The α-Wasserstein GAN (α-WGAN)^11^ and cycle consistent embedding GAN (CCE-GAN)^10^ models rely on an encoder–decoder architecture in which images are encoded into a latent space and then decoded; additional synthetic images can then be decoded from random noise. This architecture aims to bring the real image latent representation close to random noise, such that after training randomly sampled noise will be decoded into meaningful images. On the other hand, HA-GAN^12^ relies on having small-resolution and slice-based high-resolution generators and discriminators. The generators and discriminators partially share weights, thus enabling high-resolution sampling during inference. α-WGAN and CCE-GAN lack any form of conditioning while HA-GAN^12^ includes class-based conditioning. These models present either basic or non-existing conditioning, but none have quantified how morphologically persistent the synthetic samples are—a paramount trait if we are to use such methods.

The most popular application of generative models in medical imaging is data augmentation, mainly for classification and segmentation tasks^15^, with the aim of increasing the diversity of the phenotype by grounding the generative process onto a ground truth segmentation or inpainting segmentations of different pathologies onto healthy subjects from the training dataset^16^. However, none of those methods truly expand the healthy phenotype manifold or add pathological phenotypes. A fully unsupervised 2D generative model of both human brain parcellations and corresponding images has recently been reported^17^.

A subject’s phenotype is determined by several covariates, such as demographic characteristics and the presence or absence of pathologies. When analysed together, multiple subjects with the same covariates of interest will determine a population-level morphological statistic. In line with the synthetic data desiderata from ref. ^18^, any usable synthetic dataset should share most, if not all, the statistical properties of the real one. Towards that end, a synthetic model must have a controllable sampling mechanism. While current state-of-the-art generative models of natural images^19,20^ have demonstrated astonishing control over the generation of synthetic samples, their ability to preserve the morphology of the generated objects remains to be validated. Synthetic images are often validated using qualitative human evaluation and relatively simple classifier-based metrics such as the Frechet inception distance (FID)^21^, which are insufficient when applied to medical data. In ref. ^22^ image moments were used to asses pixel distribution alignment and a reader study was used to assess the synthetic sample realism of medical data; however, no quantitative morphological analysis was performed. Without validating the preservation of patient and disease morphology, one would not be able to trust downstream data use and subsequent analysis.

**Fig. 1:**
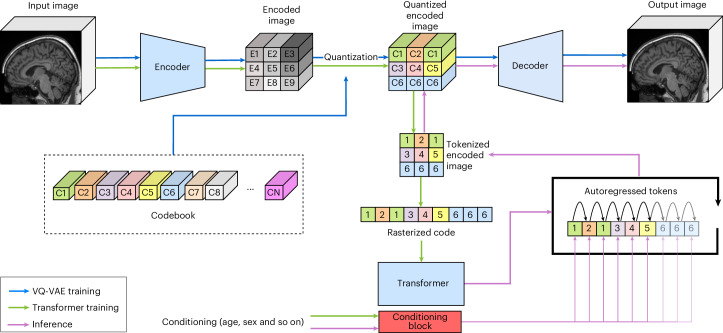
Pipeline architecture. The VQ-VAE and transformer two-stage training and inference pipeline is shown. During the VQ-VAE training (blue arrow) a Codebook representation is learned in order to minimise the reconstruction loss between the Input image and Output image. For stability a consistency loss is applied to the Codebook elements in regards to the Encoded image. For the Transformer training the autoregressive conditional generation is learned via a cross-entropy loss applied to the Rasterized code. For inference the Transformer is conditioned on the variables of interest and generates on token at a time, once the whole sequence is generated it is reshaped into a Tokenized encoded image and fed through the Codebook to the Decoder in order to obtain the Output image.

In natural image synthesis, the combination of a vector-quantized VAE (VQ-VAE) and transformer has recently^23,24^ been shown to generate high-resolution realistic images. They work by projecting an image into a quantized space where the transformer then learns the conditional probability of tokens in an autoregressive fashion. Afterwards, the trained transformer can synthesize new latent samples and pass them to the VQ-VAE to be decoded. In our previous work^25,26^ we have shown that the pipeline depicted in Fig. 1 can be used in clinical settings for anomaly detection, which represents a much more locally restricted task of token inpainting. In this work, we show that such models can be scaled up to generate realistic-looking morphologically preserving synthetic samples. To assess their realism, we use classic metrics such as the FID^21^ and maximum mean discrepancy (MMD)^27^ with image diversity being assessed through the multi-scale structural similarity index measure (MS-SSIM)^28^ and four-gradient-structural similarity index measure (4-G-SSIM)^29,30^ as used in ref. ^11^. To study the morphology of the generated data, we compare the tissue segmentations and subcortical volumes generated by SynthSeg^31^ between the synthetic and real samples, assess the distribution alignment between cortical thicknesses of synthetic and real samples as measured by FastSurfer^32^ and look at the focal difference between real and synthetic subpopulations using voxel-based morphology (VBM)^33^. We will make both the trained models and code available to the research community, together with two sampled synthetic datasets: a UK Biobank (UKB)^4^ based dataset of 100,000 healthy participants and 1,000 pathological and cognitively normal participants based on Alzheimer’s Disease Neuroimaging Initiative (ADNI)^6^.

## Results

We used two datasets, the UKB dataset formed of 39,679 neurologically healthy participants and the ADNI dataset, which encompasses 765 unique participants. Further details on the datasets, preprocessing and augmentation can be found in Supplementary Section A.

The methods we are comparing against are the state-of-the-art medical imaging generative models Hierarchical Amortized GAN (HA-GAN)^12^, CCE-GAN^10^ and Least Squares GAN (LS-GAN)^34^. In the ‘Quantitative image fidelity evaluation’ section we present a quantitative evaluation of their realism and in the ‘Morphological evaluation’ section we assess the morphological correctness of the synthetic samples. Finally, we provide a detailed ablation study of our pipeline, showcasing how each design’s choice and model’s scale influenced the results. This can be found in Supplementary Section B.

All baselines underwent a 25 experiment grid search to optimize them on our datasets and were trained on one NVIDIA A100 DGX Superpod with the maximum batch size possible for 20,000 iterations. In line with ref. ^12^, CCE-GAN was modified to work at an image size of 128^3^ and its results were upsampled. The same augmentation pipeline was applied to all models, while the intensity thresholding and normalization transformations were configured per baselines’ official implementations. The *T*_1_*w* VQ-VAE models were trained on one NVIDIA A100 DGX Superpod, while the FLAIR and ${T}_{2}^{\,* }$ VQ-VAE models that were trained for the ‘Quantitative image fidelity evaluation’ section generalizability were trained on a single NVIDIA V100 32 GB card. The small transformers were trained on four NVIDIA A100 DGX Superpods while the big transformers were trained on eight NVIDIA A100 DGX Superpods.

### Quantitative image fidelity evaluation

**Fig. 2:**
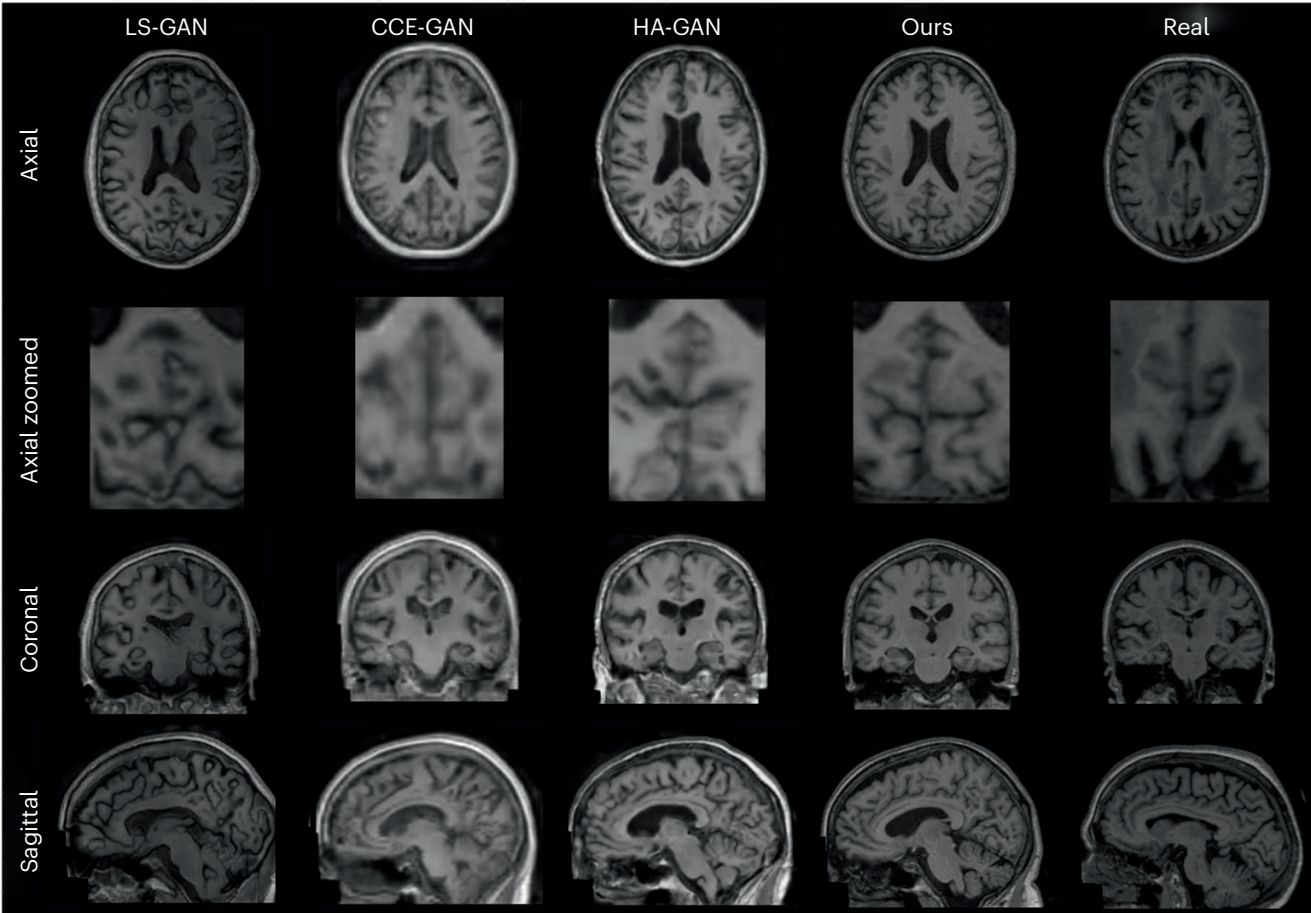
Random synthetic samples from ADNI dataset. Random synthetic samples from the LS-GAN, CCE-GAN and HA-GAN baseline trained on ADNI together with our proposed model trained on ADNI and the a real participant from the datasets. All three planes of visualization (axial, coronal and sagittal) are presented. Additionally an axial zoomed in visualization of the cerebellum is showcased due to the higher number of cortical folds entailing more high-frequency details. All the visualization planes are from the same synthetic samples and real participant.

**Extended Data Fig. 1:**
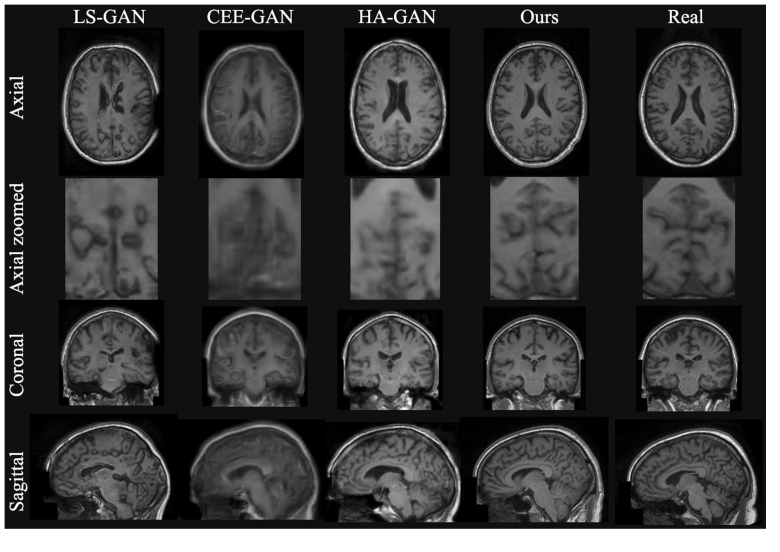
UKB samples from each baseline model, the proposed model, and the real data. Random synthetic sample from each model trained on UKB as well as the real dataset.

To evaluate the sample realism, we trained the models on all of the available data and, where possible, we sampled in a controlled manner. The synthetic datasets had the same number of samples as their real counterparts to guarantee a fair comparison. As shown in Table 1, the proposed model outperformed all of the baselines by a wide margin, ranging up to two orders of magnitude in the case of FID and MMD. The diversity measured by the MS-SSIM and 4-G-SSIM was roughly the same. This should be considered together with the poor image sampling quality displayed in Extended Data Fig. 1 and Fig. 2 for other baselines. Altogether, our model generated sharper images that better adhere to the dataset’s underlying distribution. The HA-GAN sampling quality came closest to the proposed models, but it had apparent artefacts within the white matter, which did not align with the real morphology as showcased in the ‘Morphological evaluation’ section. While LS-GAN provided better FID than CCE-GAN, it lacked distribution alignment, as shown by the MMD. This discrepancy can be attributed to CCE-GAN working on 128^3^ downsampled space, which was then upsampled for quantitative metric calculations. Furthermore, manual inspection revealed that LS-GAN shows signs of mode collapse due to its reduced diversity in sampling compared with CCE-GAN.

**Table 1:** Quantitative evaluation of the image fidelity and diversity of synthetic samples

Dataset	Model	FID^a,^^b^	MMD	MS-SSIM^c^	4-G-SSIM
UKB	Ours	0.0026	9.53 × 10^−7^	0.67 ± 0.05	0.40 ± 0.02
UKB	HA-GAN^12^	0.0047	1.4 × 10^−6^	0.66 ± 0.08	0.42 ± 0.06
UKB	CCE-GAN^10^	0.0888	4.08 × 10^−5^	0.66 ± 0.05	0.38 ± 0.01
UKB	LS-GAN^34^	0.0171	1.26 × 10^−6^	0.65 ± 0.04	0.38 ± 0.01
ADNI	Ours	0.0075	5.21 × 10^−7^	0.69 ± 0.07	0.40 ± 0.06
ADNI	HA-GAN^12^	0.0219	1.99 × 10^−6^	0.59 ± 0.09	0.38 ± 0.06
ADNI	CCE-GAN^10^	0.1680	5.96 × 10^−5^	0.59 ± 0.06	0.37 ± 0.03
ADNI	LS-GAN^34^	0.0763	8.86 × 10^−6^	0.59 ± 0.05	0.37 ± 0.2

^a^A lower FID and MMD indicate better distribution alignment between the real and synthetic samples, while a lower MS-SSIM and 4-G-SSIM indicate higher diversity of synthetic samples.

^b^FID and MMD are lower bounded by 0 with no upper bound while MS-SSIM and 4-G-SSIM are lower bounded by 0 with an upper bound of 1.

^c^For MS-SSIM and 4-G-SSIM the mean and standard deviations are presented.

**Extended Data Fig. 2:**
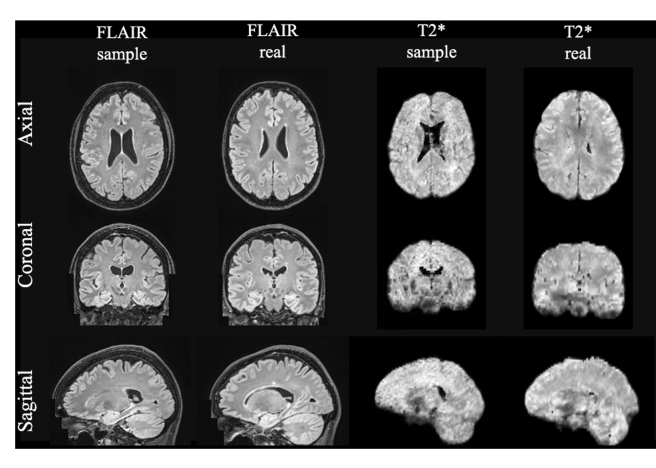
UKB samples from the proposed model trained on T2* and FLAIR data. Random synthetic sample from our model trained on UKB’s T2* and FLAIR as well as corresponding real samples.

To gauge how well the pipeline generalized, we trained a big VQ-VAE and a small transformer on the ${T}_{2}^{\,* }$ and FLAIR images from the UKB dataset. Similarly, we sampled the same number of synthetic samples as their real counterparts to guarantee a fair comparison. As shown in Table 2 and Extended Data Fig. 2, the pipeline showed generalizability potential when looking at the FLAIR results but underperformed with ${T}_{2}^{\,* }$ images. The main culprit of the difference in performance was the difference in the scaling of the VQ-VAE between the *T*_1_ weighted and other models. This manifested as an underutilized latent representation as measured by the average perplexity of the VQ-VAE’s codebook elements between the ${T}_{2}^{\,* }$ and FLAIR models when compared with the *T*_1_ weighted one. Quantitatively, this resulted in 4-G-SSIM values of 0.392 ± 0.019 and 0.513 ± 0.024 for ${T}_{2}^{\,* }$ and FLAIR, respectively. A manual inspection showed that this resulted, from a qualitative point of view, in a lower cortical fold diversity for the FLAIR model. In the case of the ${T}_{2}^{\,* }$ model, as observed in Extended Data Fig. 2, the VQ-VAE was unable to model the super-resolved anisotropic images. The ${T}_{2}^{\,*}{{{\rm{s}}}}$ images were originally acquired at a voxel resolution of 0.76 mm × 0.76 mm × 1 mm and super-resolved during preprocessing to 1 mm × 1 mm × 1 mm. Overall, with additional improvements to the codebook update strategy, this pipeline should yield good generalizability.

**Table 2:** Evaluation of the image fidelity and diversity of the synthetic samples across multiple modalities

Model	Model	FID	MMD	MS-SSIM	4-G-SSIM
*T*_1_ weighted	Transformer	0.0026	9.53 × 10^−7^	0.67 ± 0.05	0.40 ± 0.02
${T}_{2}^{\,*}$	Transformer	268.72	0.0226	0.81 ± 0.03	0.49 ± 0.03
FLAIR	Transformer	0.679	2.55×10^−4^	0.68 ± 0.04	0.36 ± 0.02

### Morphological evaluation

#### VBM

To assess whether the focal differences between population subgroups were preserved in the synthetic data, we used VBM^33^ as implemented in the SPM software package^35^ (https://www.fil.ion.ucl.ac.uk/spm/). VBM identifies the morphological differences in modulated tissue compartments between group-aligned selected groups through a generalized linear model and associated statistical tests across all voxels. For a detailed description of how VBM works, please see ref. ^36^. In line with ref. ^37^, all *t*-statistics maps were corrected to minimize the spurious effects of low-variance areas. In this experiment, two datasets were created. First, for the healthy dataset, we aimed to assess the morphological differences between a small ventricle population and a big ventricle population, defined on the basis of the ventricular cerebrospinal fluid (CSF) segmentation. This experiment was chosen as the expected pattern of differences should be trivial and localized in the ventricular region. Specifically, the small ventricle population was formed of 160 random participants from the first quintile, while 160 subjects formed the big ventricle from the last (fifth) quintile. Second, for the pathological dataset, we evaluated the differences between 145 cognitively normal participants and 185 with Alzheimer’s disease, defined by their clinical diagnoses; the grey matter segmentation was chosen to visualize the difference between populations.

Contrary to the proposed method, which was conditioned on the actual ventricular volume size, HA-GAN was conditioned on a discretized class of ventricular sizes as determined by a 5-quantile of the healthy dataset and on the cognitively normal/Alzheimer’s disease binary label for the pathological dataset. This is due to the limitation of HA-GAN, which requires class-based conditioning. While our model and HA-GAN were sampled in a controlled manner, LS-GAN and CCE-GAN do not allow for conditioning, so a separate model was explicitly trained for each population. Note that due to the conditional sampling capabilities of our model, its VBM analysis factors out age and sex. The template scripts used for VBM can be found in Supplementary Section D.

**Fig. 3:**
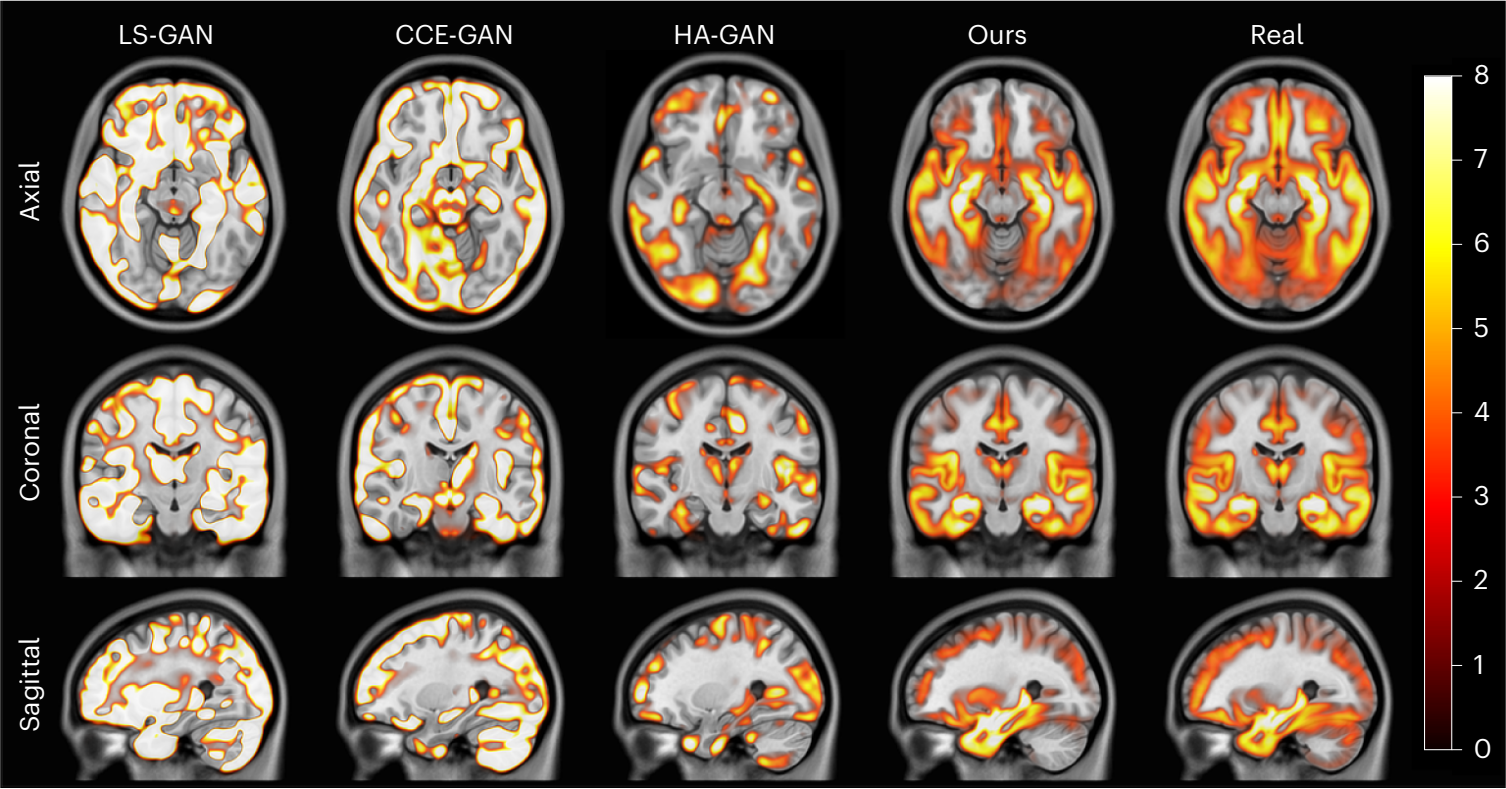
VBM *t*-statistics maps of grey matter for the ADNI pathological dataset. Maps for all models and real data are shown. The displayed *t*-statistics range is [0, 8] (colour scale) and is based on the VBM of the real data. The *t*-statistics were corrected following the procedure used in ref. ^37^.

**Extended Data Fig. 3:**
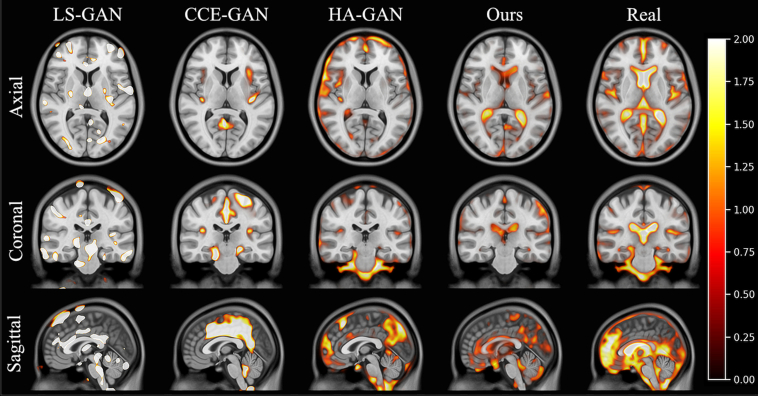
VBM t-statistic maps of cerebrospinal fluid for UKB healthy dataset from the baseline models, proposed model and real data. VBM t-statistic maps of cerebrospinal fluid for UKB healthy dataset. The displayed t-statistics range is [0, 2] and is based on the VBM of the real data. The t-statistics were corrected as per^36^.

Extended Data Fig. 3 and Fig. 3 show that the proposed model is in significantly better agreement with the real data than competing methods. As shown in Extended Data Fig. 3, for the healthy dataset, our model captured the differences in the ventricles, subgenual area and partially in the left and right insulae. However, HA-GAN could not sample in a controlled manner and could not model the ventricular size, and also overemphasized the CSF between the meninges and the brain. LS-GAN and CCE-GAN were unable to properly model the brain’s structure, resulting in uninformative VBM maps. Our model, however, as shown in Fig. 3, was in near-perfect agreement with the real data for the pathological dataset as it captured the overall morphological differences in the putamen, hippocampal area, temporal gyrus, inferior occipital gyrus and fusiform gyrus. Even though the other baselines seem to capture the morphological differences, they were more in line with the general atrophy of the brain due to ageing, lacking the focal increase in *t*-statistics showcased in the real data.

#### Subcortical volume analysis

To further validate the morphology of the data, we compared regional volumes between real and synthetic data as estimated by SynthSeg^31^ (https://github.com/BBillot/SynthSeg). All images were segmented into grey matter, white matter and CSF. Table 3 shows that the proposed model better adhered to the original tissues’ volumetric distribution than other baselines. We also ran a two-sided Mann–Whitney *U*-test^38^ between the models’ segmentation distributions and the real ones and Glass’s Δ effect size. We evaluated the distribution shift through the Wasserstein distance and Kullback–Leibler divergence. The results in Table 3 and Extended Data Table 1 show that our model’s synthetic samples better adhered to the real distribution. This can be seen by looking at the Wasserstein distance and Kullback–Leibler divergence specifically, for which our model and HA-GAN had the best values across all datasets. The UKB results show that our model outperformed the HA-GAN model, only underperforming on the Wasserstein distance of the CSF tissue. On the ADNI dataset, HA-GAN showed superior performance that could be attributed to the lack of diversity in our model, as seen in the MS-SSIM and 4-G-SSIM values in Table 1.

**Table 3:** Tissue volumes based on SynthSeg segmentations

Dataset	Model	Grey matter	White matter	CSF
UKB	Real	568.02 (40.30) mm^3^	473.03 (44.97) mm^3^	373.85 (37.90) mm^3^
UKB	Ours	562.02 (35.74) mm^3^	475.88 (40.61) mm^3^	363.05 (26.12) mm^3^
UKB	HA-GAN^12^	544.14 (33.86) mm^3^	465.62 (45.49) mm^3^	383.08 (23.84) mm^3^
UKB	CCE-GAN^10^	407.31 (130.66) mm^3^	388.15 (100.04) mm^3^	323.31 (56.13) mm^3^
UKB	LS-GAN^34^	442.84 (1.38) mm^3^	413.42 (1.15) mm^3^	372.65 (1.35) mm^3^
ADNI	Real	510.58 (55.04) mm^3^	425.43 (52.75) mm^3^	418.88 (40.42) mm^3^
ADNI	Ours	513.21 (33.10) mm^3^	442.91 (31.91) mm^3^	402.04 (19.69) mm^3^
ADNI	HA-GAN^12^	505.91 (36.25) mm^3^	441.22 (35.06) mm^3^	405.43 (27.22) mm^3^
ADNI	CCE-GAN^10^	502.08 (17.89) mm^3^	428.17 (8.81) mm^3^	400.45 (17.65) mm^3^
ADNI	LS-GAN^34^	439.70 (1.68) mm^3^	404.96 (1.39) mm^3^	419.97 (3.08) mm^3^

Data are presented as the median with the interquartile range in parentheses. For ease of reading the volume is presented as a multiple of 10^3^.

As downstream tasks do not rely solely on tissue statistics, we also analysed the individual subcortical volumes of the SynthSeg parcellations (Extended Data Table 2). We ranked the models by calculating the average rank across all subcortical regions as measured by the Wasserstein distance between the synthetic and real populations. As can be seen in Extended Data Table 2, our method had the best rank across UKB and ADNI datasets. Those results, combined with the VBM results in Extended Data Fig. 3 and Fig. 3, show that our model had better global and local morphological preservation.

Given that downstream tasks can silently fail by giving results that do not adhere to the expected behaviour, we quantified the failure rate of the SynthSeg pipeline. This was done by measuring the proportions of the synthetic samples with at least one region with an absolute *Z* score greater than 5, meaning that they fall outside the 99.99th percentile. For any region, a failure rate above 0.01% can be attributed to the model’s performance. The results for the SynthSeg pipeline can be found in Extended Data Table 2; our model outperformed all of the baselines as it has closest failure rate to real samples. A failure rate lower than real data can be explained by the model not covering the extreme modes of the real datasets that fall outside the 99.99th percentile. An extreme case of this can be seen for the CCE-GAN model, which achieved a 0% failure rate. On the other extreme is LS-GAN that, from the quantitative results in Table 1, achieved a 100% failure rate.

#### Cortical thickness analysis

Cortical thickness estimation is one of the most sensitive biomarkers that can be used to assess a wide range of brain conditions, ranging from ageing to neurological disorders^39^. We used FastSurfer^32^ (https://github.com/Deep-MI/FastSurfer) on 100 randomly selected cognitively normal participants from the ADNI dataset to estimate the mean cortical thickness. FastSurfer is a convolutional neural network-based surface-based thickness analysis tool that parcellates and quantifies the cortical thickness of 32 regions per hemisphere. We used the same methodology as in the ‘Subcortical volume analysis’ section for the FastSurfer cortical parcellations. As can be seen in Extended Data Table 3, our model had the best performance and the closest failure rate to the real data. Having the best rank in both Extended Data Table 2 and Extended Data Table 3 could be attributed to better modelling of the variability between participants and structural coherence. Those results further reinforce the morphological preservation capabilities of our model. Interestingly, LS-GAN had a better failure rate than HA-GAN despite not performing morphologically or quantitatively as well.

#### Train on synthetic, test on real analysis

**Extended Data Fig. 4:**
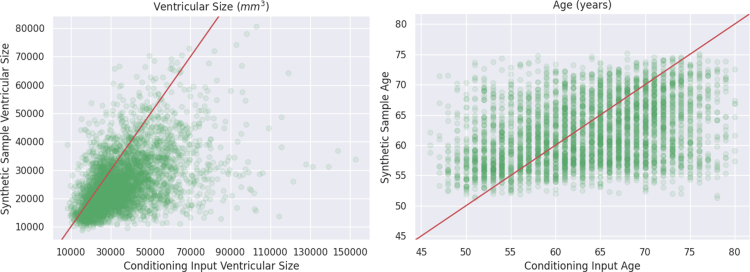
Visualization of the predicted sampled conditioning vs the input conditions. Correlation between the measured and the conditioning variable, when applied to Age and Ventricular size. The Pearson Correlation of Ventricular Size and Age is 0.47 and 0.33 respectively, while the p-Value is 6.71E-21 and 3.51E-102.

We trained a simple fully convolutional network (SFCN)^40^ and used it to predict the age of the synthetic samples and the SynthSeg segmentations to calculate the ventricular size of the synthetic samples. Afterwards, we calculated the Pearson correlation coefficient between the input conditioning of the given samples and the regressed age and ventricular size. The correlations and results are illustrated in Extended Data Fig. 4. Looking at the correlations between the conditional age versus the predicted age and the conditional ventricular size versus the SynthSeg calculated ventricular size we found a statistically significant (*P* < 0.001, 0.0005 Bonferroni corrected), but still relatively weak, correlation of 0.37 for age and a moderate correlation of 0.47 for ventricular size. We also found that smaller transformer models had poorer correlations during internal testing, suggesting that model capacity might limit performance.

We quantified the clinical viability and usefulness of the proposed model following the train on synthetic, test on real paradigm outlined in ref. ^41^. The SFCNs^40^ were cross-validated to regress age for the UKB dataset. Each SFCN’s parameters were first optimized and evaluated on the synthetic samples. Afterwards, a new model with the same parameters was trained and evaluated on the real dataset. Each synthetically trained model was evaluated on the full real data to assess its generalizability capability without any fine-tuning. While the SFCN model trained on the real data and tested on real data achieved a mean absolute error (presented as mean ± s.d.) for regressing the age of 3.05 ± 2.47 yr, the model trained on synthetic and tested on synthetic data achieved a mean absolute error of 4.05 ± 3.74 yr. When the synthetic-trained SFCN was tested on real data, it achieved a mean absolute error of 5.34 ± 3.73 yr. The proposed model shows promise for application within the training on synthetic, testing on real paradigm^41^. The SFCN model trained on synthetic healthy UKB data generated by our model had results comparable to those of the model trained on the real dataset. The drop in performance could be attributed to the degree of compression needed for the transformer and the correlations that the conditionings have, as shown in Extended Data Fig. 4. This puts a lower-bound limit on the performance that a synthetically trained model can have with the real data.

## Discussion

In this work we developed a deep generative model capable of creating morphologically preserving realistic 3D images of the brain. The quantitative analysis shows that the proposed model achieved state-of-the-art performance in image synthesis. At the same time, the voxel-based morphology, together with subcortical and cortical analyses, demonstrated the superior morphological preservation of our model. Our training on synthetic, test on real experiments suggests that synthetic data could one day replace real data for AI model training in privacy-sensitive fields such as healthcare. The main limitations of this work were the application of quantitative and morphological analysis to a single organ, the brain, and to a single diagnosis tool, the MRI scans. We hope that this work paves the way towards a more principled evaluation of synthetic data in the field of healthcare, and more specifically brain imaging, by incorporating morphological analyses alongside the classical quantitative ones.

## Methods

First, we review how the VQ-VAE and transformer pipeline works. Following that we introduce our VQ-VAE and the transformer. Lastly, we detail the implementation of the model (see Fig. 3 for the models’ architecture).

### Background

VQ-VAEs^42,43^ can successfully synthesize high-resolution natural images^23,44^. VQ-VAEs are composed of an encoder Enc that takes as input an image $X\in {{\mathbb{R}}}^{H\times W\times D}$ and projects it to a smaller latent representation $Z\in {{\mathbb{R}}}^{h\times w\times d\times {n}_{z}}$ where *H*, *W*, *D* and *h*, *w*, *d* are the height, width and depth of the input image and latent representation respectively with *n*_*z*_ being the dimensionality of the latent embedding’s vector. Afterwards, *Z* is passed through the quantization block Quant where an element-wise quantization is done. Each spatial code ${Z}_{ijk}\in {{\mathbb{R}}}^{{n}_{z}}$ is replaced by its nearest codebook element ${{e}}_{m}\in {{\mathbb{R}}}^{{n}_{z}},m\in 1,\ldots ,M$ where the vocabulary’s size is denoted *M*, thus obtaining *Z*_*q*_, the quantized representation. The codebook’s elements are learned in an online fashion through exponential moving average (EMA) as part of the VQ-VAE training procedure. Given *Z*_*q*_, the decoder Dec tries to reconstruct the observations $\hat{X}\in {{\mathbb{R}}}^{H\times W\times D}$. This is outlined by the blue flow path in Fig. 3.

In the second stage, a generative model Gen is trained on the latent discrete representation. The representation is obtained by replacing the codebook elements of *Z*_*q*_ with their respective indices, thus obtaining *Z*_i*q*_. In refs. ^42,43^, a PixelSNAIL^45^ autoregressive model was originally employed. Later, ref. ^23^ showed improved performance by replacing PixelSNAIL with a transformer^46^. As transformers work on sequences, *Z*_i*q*_ is flattened in a row-major fashion, thereby obtaining *S*_i*q*_. The transformer is then used to model *S*_i*q*_ by minimizing the conditional distribution $p({{S}_{{\mathrm{i}}q}\!}_{j+1})=p({{S}_{{\mathrm{i}}q}\!}_{j}| {{S}_{{\mathrm{i}}q}}_{ < j},c)$ where *p* is probability, ${{S}_{{\mathrm{i}}q}\!}_{j}$ is the *j*th element of *S*_i*q*_ and *c* are conditioning variables. The green flow path in Fig. 3 shows this, together with an on-the-fly augmentation by passing augmented images through the encoder and quantization before flattening, such that *S*_i*q*_ benefits from the augmentation.

The transformer^46^ is composed of multiple layers, each equipped with an (self-)attention mechanism. The attention mechanism can capture the interaction between the elements of *S*_i*q*_ elements regardless of their relative position to each other. This is achieved by projecting the intermediary representation into three vectors: query, key and value; and can be written as follows:

1 $${\mathrm{Attention}}(Q,K,V)={\mathrm{softmax}}\left(\frac{Q{K}^{T}}{\sqrt{{d}_{K}}}\right)V$$

where *T* stands for transpose, *Q* is the query tensor, *K* is the key tensor, *V* is the value tensor and *d*_*K*_ is the dimension of *K*.

The projection happens once per head, where each head will learn to attend to different concepts of the sequence. During training, the model aims to predict each of ${{S}_{{\mathrm{i}}q}\!}_{j}$ based on ${{S}_{{\mathrm{i}}q}}_{ < j}$ and *c*. This happens due to the autoregressive training method, in which the attention mechanism is masked such that it only attends to the elements before it. The conditioning is applied at every other attention block where the inputs to the key and value layers are replaced by a vector formed from the concatenation of the arbitrary conditionings. This approach is known as cross-attention and allows the network to attend to the conditionings offered, thus enabling controlled sampling during inference^46,47^.

At inference, the model predicts the probability distribution of a single token at a time $p({{S}_{{\mathrm{i}}q}\!}_{j}| {{S}_{{\mathrm{i}}q}}_{ < j},c)$. On the basis of the estimated probability, it randomly picks one of the tokens. This procedure is applied for each token location sequentially until the full sequence is sampled. Afterwards, *S*_i*q*_ is reshaped back to a 3D tensor into *Z*_i*q*_ and passed through the quantizer to obtain *Z*_*q*_, which is then decoded into the image space. This step of the pipeline is represented by the purple flow line in Fig. 3.

### Descriptive quantization for transformer use

For the transformer to synthesize meaningful samples, the VQ-VAE’s latent representation needs to meet the following two requirements: it should be small enough to meet the memory constraints of the transformer architecture and be sufficiently descriptive such that the reconstruction is both structurally coherent and realistic.

Towards the first requirement, our VQ-VAE projects the input image from a tensor $X\in {{\mathbb{R}}}^{160\times 224\times 160}$ to ${Z}_{{\mathrm{i}}q}\in {{\mathbb{N}}}_{0}^{10\times 14\times 10}$. This compresses the image spatially by a factor of 4,096, or 14,564 if we take into account the data types conversion from floating point 32 to integer 8. This results in a sequence *S*_i*q*_ of length 1,400, which is sufficiently small to be modelled by a transformer. *Z*_*q*_ was originally learned by gradient descent as follows:

2 $${{{{\mathcal{L}}}}}_{\mathrm{quant}}(X\,)=\left\Vert{\mathrm{sg}}[Z\,]-{Z}_{q}\right\Vert_{2}^{2}+\beta\left\Vert{\mathrm{sg}}[{Z}_{q}]-Z\right\Vert_{2}^{2}$$

where sg is the stop-gradient operation. As per refs. ^42,43^, the second component in equation (2) is replaced by equation (3), where ${n}_{\mathrm{i}}^{(t)}$ is the number of vectors in *Z* that will be quantized at exponential moving average update timestep *t* to the codebook element ${{Z}_{q}}_{\mathrm{i}}$. The hyperparameters *γ* and *β* control the decay of the EMA from equation (3) and the commitment of the encoder output to a certain quantized element, respectively. This changes the learning procedure of *Z*_*q*_ from a gradient-descent-based one to an online EMA procedure.

3 $$\begin{array}{l}{N}_{i}^{(t)}:= {N}_{i}^{(t-1)}\times \gamma +{n}_{i}^{(t)}(1-\gamma ),\quad {m}_{i}^{(t)}:= {m}_{i}^{(t-1)}\times \gamma\\\qquad\quad+\sum\limits_{j}^{{n}_{i}^{(t)}}{Z}_{i,\,j}^{\,(t)}(1-\gamma ),\quad {{{Z}_{q}}_{i}}^{(t)}:= \frac{{m}_{i}^{(t)}}{{N}_{i}^{\,(t)}}\end{array}$$

To meet the second requirement (that is synthetic samples being structurally coherent and realistic), significant change to the VQ-VAE loss was necessary to appropriately cater to and stabilize the training on 3D medical data. We started with the classical mean squared error (MSE) ${{{{\mathcal{L}}}}}_{pix}=MSE(X,{\hat{X}}\,)$ that works purely on the pixel domain. We took inspiration from ref. ^48^ and used the MSE on the amplitude of the fast Fourier transformation of *X* and $\hat{X}$, which can be written as ${{{{\mathcal{L}}}}}_{freq}=MSE(| FFT(X\,)| ,| FFT(\hat{X}\,)| )$ to improve the sharpness of the reconstructions. Following that, we added a perceptual loss based on the LPIPS^49^ package using AlexNet^50^ as the feature extractor. Note that the LPIPS pre-trained network is 2D, so owing to the 3D nature of medical images this loss was applied on a slice-wise basis. More specifically, we applied it to 50% of randomly selected slices of each axis, resulting in:

4 $${{{{\mathcal{L}}}}}_{\rm{pcp}}={{{\mbox{LPIPS}}}}_{0.5}({X}_{\rm{ax}},{\hat{X}}_{\rm{ax}})+{{{\mbox{LPIPS}}}}_{0.5}({X}_{\rm{sag}},{\hat{X}}_{\rm{sag}})+{{{\mbox{LPIPS}}}}_{0.5}({X}_{\rm{cor}},{\hat{X}}_{\rm{cor}})$$

where ax, sag and cor representing the axial, sagittal and coronal planes, respectively.

With LPIPS loss we aimed to increase the perceived quality, as well as increasing the convergence, similarly to the reasoning presented in ref. ^51^. Finally, we used an adversarial loss as the images showed intensity patterns that were not realistic:

5 $${{\mathcal{L}}}_{\rm{adv}}={{\mathcal{L}}}_{\rm{LSGAN}}(Dis)+{{\mathcal{L}}}_{\rm{LSGAN}}(Enc,Quant,Dec)$$

This is based on the Patch-GAN^52^ model as per ref. ^23^ paired together with the LS-GAN loss^34^, and provided more stable and reproducible behaviour, which can be written as:

6 $$\begin{array}{rcl}\mathop{\min}\limits_{Dis}{L}_{{{{\rm{LSGAN}}}}}(Dis)&=&\displaystyle\frac{1}{2}{{\mathbb{E}}}_{{{{\bf{x}}}} \sim {p}_{{{{\rm{data}}}}}({{{\bf{x}}}})}\left[{(Dis(X\,)-1)}^{2}\right]\\ &&+\displaystyle\frac{1}{2}{{\mathbb{E}}}_{{{{\bf{x}}}} \sim {p}_{{{{\bf{data}}}}}({{{\bf{x}}}})}\left[\left.\right(Dis{(D(Q(E(X\,))))}^{2}\right]\\\displaystyle\mathop{\min}\limits_{Enc,Q,Dec}{L}_{{{{\rm{LSGAN}}}}}(E,Q,D)&=&\frac{1}{2}{{\mathbb{E}}}_{{{{\bf{x}}}} \sim {p}_{{{{\rm{data}}}}}({{{\bf{z}}}})}\left[{(Dis(D(Q(E(X\,))))-1)}^{2}\right]\end{array}$$

A quantitative assessment of the effect of and need for these metrics is presented in the ablation study in Supplementary Section B. Overall, the proposed VQ-VAE loss function is:

7 $${{{{\mathcal{L}}}}}_{\mathrm{VQ-VAE}}={{{{\mathcal{L}}}}}_{\mathrm{quant}}+{\alpha }_{\mathrm{pix}}* {{{{\mathcal{L}}}}}_{\mathrm{pix}}+{\alpha }_{\mathrm{freq}}* {{{{\mathcal{L}}}}}_{\mathrm{freq}}+{\alpha }_{\mathrm{pcp}}* {{{{\mathcal{L}}}}}_{\mathrm{pcp}}+{\alpha }_{\mathrm{dis}}* {{{{\mathcal{L}}}}}_{\mathrm{dis}}$$

### Autoregressive modelling of the brain

Clinically usable, fully unsupervised generative modelling of medical images should preserve morphology. To ensure that our synthetic samples do so, we enhanced a baseline transformer implementation from refs. ^42,43^ with a series of techniques that have been tailored to facilitate controlled sampling sequences that also have whole-sequence contextual information as well as 3D inductive bias. Furthermore, one of the techniques^53^ has been generalized from 2D to 3D. In the rest of this section we detail each of the techniques and the reasons we chose it.

As the transformer takes a 1D sequence as input *S*_i*q*_, it loses the intrinsic spatial inductive bias of *Z*_i*q*_. To reintroduce it, we implemented a 3D generalization to the relative positional bias^53^ approach. It works by adding a bias term to the **QK** vector pre-softmax in the attention mechanism. The bias term is based on the product of the directional relative positional embedding of each voxel. As we were working on a full latent representation we did not quantize the distance with a piecewise function as done by ref. ^53^ because our *Z*_i*q*_ had 46 times fewer spatial positions than an ImageNet sample. This is due to the high compression rate that was achieved with the VQ-VAE.

Thus, the transformer’s hidden state should contain contextual information about the whole sequence. For that, we introduced an enhanced recurrence, similarly to the procedure in ref. ^54^. It is a simple mechanism that passes the outputs of the next layer from the previous sampling step to the current layer in the current sampling step. Furthermore, it enhances future lower-level representation with higher-level representations.

Owing to the size of the sequence and the scaling requirements, we employed root mean squared error normalization^55^. This was shown to be the best-performing normalization variant for transformers in ref. ^56^ and was used in large language models such as those in refs. ^57,58^. We noticed that this approach was of paramount importance for the models to converge in a reasonable amount of time.

In line with ref. ^59^ and to enhance our dataset size, we augmented the samples fed into the transformer with the same augmentation protocol used during the VQ-VAE training. During training, we also conditioned our model on the random parameters sampled for the augmentations such that the model could estimate and sample during inference from the original distribution.

Finally, inspired by the success of the attention-gating mechanism in ref. ^60^, we gated our attention block outputs with the transformer’s input. This gave the model more control over each update and, in the case of cross-attention, increased the correlation between the conditioning and input.

### Implementation details

#### VQ-VAE

To showcase the fine-tuning capabilities, the value of the pre-trained models for the wider research community and in line with ref. ^61^, the UKB VQ-VAE was trained for 350 epochs for the ablation study. Following that, it was further trained for 100 epochs until convergence and then it was fine-tuned for another 100 epochs on the ADNI dataset. Adam^62^ was used as an optimizer with a learning rate of 0.000165 for the Enc and Dec networks and 0.00005 for Dis. The batch size was set to 8. The learning rates were selected via a grid search of 16 experiments. The loss components scaling factor *α*_*pix*_, *α*_*freq*_, *α*_*pcp*_ and *α*_*dis*_ were set to 1, 1, 0.001 and 1, respectively. *γ* and *β* were set to 0.25 and 0.5, respectively. The loss weights were empirically set during development. The VQ-VAE had four downsamplings with convolutions that had 128 feature maps until the last level, where they had 256. The latent representation had dimensions of 10 × 14 × 10 with a feature size of 32. Each model was trained using distributed data parallelism on an NVIDIADGX SuperPod equipped with eight A100 cards, each with 80 GB.

#### Transformer

The UKB transformer and ADNI transformer were trained for 500 epochs. Adam^62^ was used as an optimizer with a learning rate of 0.0005 and a batch size of 3. The input sequence length was the full 1,400 tokens. The transformer had 24 layers, a latent representation of 512 and 16 heads for each attention layer. Each model was trained using distributed data parallelism on four NVIDIA A100 DGX SuperPods, each equipped with eight A100 with 80 GB each. For the UKB dataset, they were conditioned on sex (UKB Data-Field 31-0.0), age (UKB Data-Field 21003-2.0), ventricular volume (UKB Data-Field 25004-2.0) and brain size normalized to head size (UKB Data-Field 25009-2.0), whereas for the ADNI dataset they were conditioned on sex, age and pathology as defined by the ARM field.

A depiction of the architecture can be seen in Fig. 3, further architectural descriptions can be found in Supplementary Section C and detailed ablation studies are elaborated on in Supplementary Section B.

### Supplementary information

Supplementary Information Supplementary Fig. 1, Tables 1–9 and Listings 1–3.

## Figures and Tables

**Extended Data Table 1:**
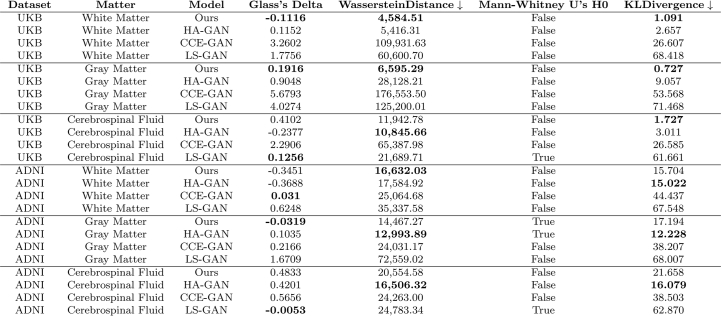
Statistical analysis of the SynthSeg results from the generative models against the real data results

The bolded values of Glass’s Delta, Wasserstein Distance and KL Divergence are the best (smallest). While the Mann-Whitney U’s results present if the null hypothesis (H0) is True or False as determined by a Bonferonni corrected statistical significance threshold of 0.001 (0.0000416).

**Extended Data Table 2:**
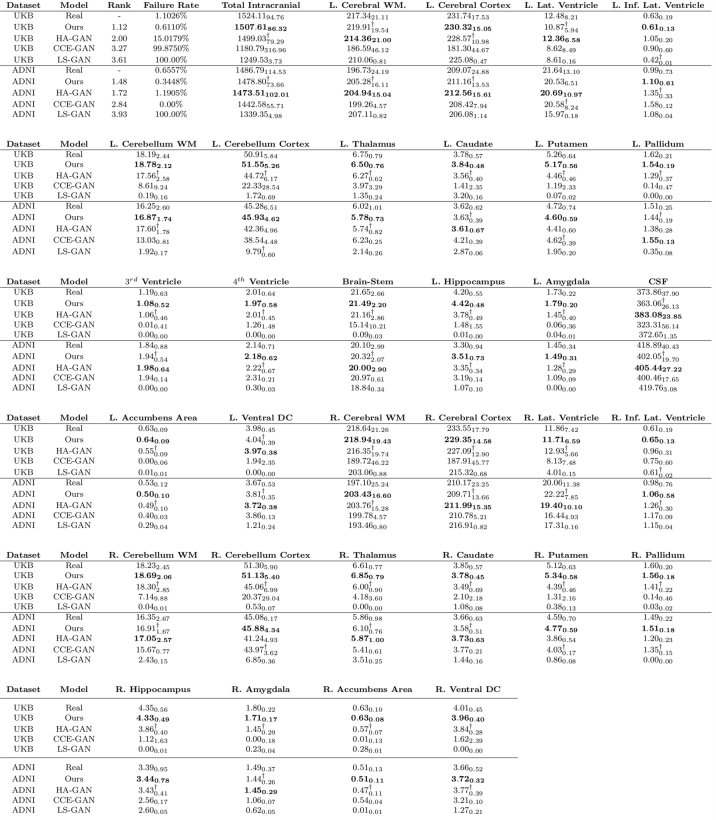
SynthSeg sub-cortical parcellation volumes from the generative models against the real data

Median and interquartile range of the SynthSeg sub-cortical parcellations (10^3^). The failure rate is calculated as the proportion of synthetic samples with at least one region with a Z-score of 5. Based on Wasserstein Distance, bolded values are closest to the real dataset while † is the second closest. Abbreviations: L. = Left, R. = Right, WM = White Matter, Lat. = Latteral, Inf. = Inferior, CSF = Cerebrospinal fluid.

**Extended Data Table 3:**
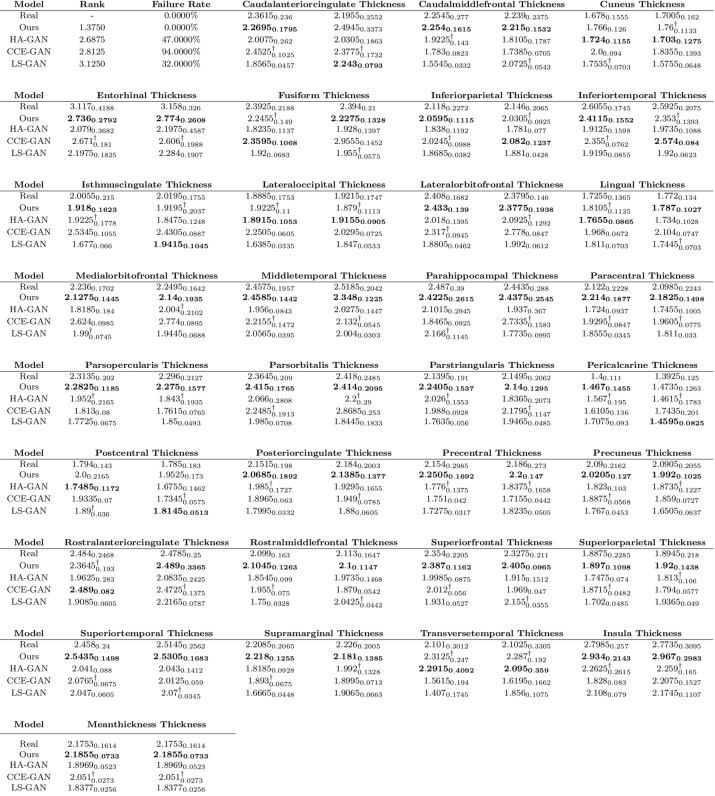
FastSurfer cortical parcellation volumes from the generative models against the real data

Median and interquartile range of the FastSurfer mean cortical thicknesses. Each cortical parcellation has a left and right sub-column for the left and right hemispheres. The failure rate is calculated as the proportion of synthetic samples with at least one region with a Z-score of 5. Based on Wasserstein Distance, bolded values are closest to the real dataset while † is the second closest.

## Data Availability

The UKB *T*_1_-weighted brain images used in this study are available via the UKB data access process (http://www.ukbiobank.ac.uk/register-apply/). Detailed information about the brain images from UKB is available at https://www.ukbiobank.ac.uk/enable-your-research/about-our-data/imaging-dataand https://biobank.ndph.ox.ac.uk/showcase/label.cgi?id=100. Given the ongoing nature of UKB study, the number of brain image samples currently available in UKB may differ slightly from those described in this Article. The ADNI *T*_1_-weighted brain images used in this study are available via the ADNI database access process (https://adni.loni.usc.edu/data-samples/access-data/). Detailed information about the brain images from ADNI is available at https://adni.loni.usc.edu/data-samples/data-types/ and https://adni.loni.usc.edu/methods/mri-tool/mri-analysis/.

## Code availability

The VBM SPM MATLAB script templates are available in Supplementary Section D. The code is open source and available via GitHub at https://github.com/AmigoLab/BrainSynth and via Zenodo at 10.5281/zenodo.11583061 (ref. ^63^).
