# Supplementary material for: Realistic morphology-preserving generative modelling of the brain
Source: Nat Mach Intell. 2024 Jul 15;6(7):811–9. doi: 10.1038/s42256-024-00864-0 (PMC11266097; doi:10.1038/s42256-024-00864-0)
Supplement: Supplementary file 1 — Supplementary Fig. 1, Tables 1–9 and Listings 1–3. [file 42256_2024_864_MOESM1_ESM.pdf]

nature machine intelligence

Supplementary information

https://doi.org/10.1038/s42256-024-00864-0

Realistic morphology-preserving generative modelling of the brain

In the format provided by the authors and unedited

Contents

A Datasets **2**

B Ablation Studies **3**

C Network Architecture Details **6**

C.1 VQ-VAE 6

C.2 Transformers 9

D Voxel Based Morphometry Details **10**

E Brain Age Model Details **15**

A Datasets

In this study, we use two datasets with T1-weighted brain images. The UKB dataset, as per our internal snapshot, consists of 39,679 neurologically healthy subjects aged between 44 and 82 years old with a mean age of 63.62±7.54 (mean±standard deviation) from which 18,691 are male subjects (47%). Their ventricular size ranges between 6,995.68 mm^3 and 171,375.00 mm^3 with a mean of 36,668.91±17,208.28 mm^3, while their brain volume normalised for head size ranges from 1,144,240 mm^3 and 1,793,910 mm^3 with a mean of 1,494,515±73,153.11 mm^3.

For the ADNI dataset we selected only the baseline appointments resulting in 765 unique subjects (148 Cognitively Normal, 25 Suspected Cognitively Impaired, 121 Early Mildly Cognitively Impaired, 282 Mildly Cognitively Impaired and 189 Alzheimer Diseased) aged between 55.16 and 91.41 years old with a mean age of 74.51±7.25 from which 432 are male (56.47%). No subsequent appointments were selected to guarantee each subject contributed equally to the manifold and to not allow subjects to transition between the different stages of Alzheimer Disease.

Both datasets have been preprocessed with UniRes[1] [1, 2] to rigidly register all data to a common MNI space. The final images were cropped, resulting in a head volume of $160 \times 224 \times 160$ voxels with a voxel size of $1mm^3$. During training, the images were min-max normalised to [0,1] and ran through an augmentation pipeline that consisted of random affine transformations, random contrast adjustments, random intensity shifts, and random The data augmentation is based on MONAI[2] version 0.5.3. and is detailed below:

**Transformation**	**Probability**	**Parameters**
ScaleIntensityd	1.0	minv=0.0, mav=1.0
CenterSpatialCropd	1.0	roi=((16,176), (16,240),(96,256))
RandAffined	0.2	rotate_range=[0.04, 0.04, 0.04], translate_range=[2, 2, 2], scale_range=[0.05, 0.05, 0.05]
RandAdjustContrastd	0.2	gamma=(0.99, 1.01)
RandShiftIntensityd	0.2	offsets=(0.0, 0.05)
RandGaussianNoised	0.2	mean=0.0, std=0.02
ThresholdIntensityd	1.0	threshold=1, above=False, cval=1.0
ThresholdIntensityd	1.0	threshold=0, above=True, cval=0.0

[1] https://github.com/brudfors/UniRes
[2] https://github.com/Project-MONAI/MONAI

B Ablation Studies

We have performed three ablation studies to showcase the effect that each contribution has on obtaining realistic synthetic samples. Firstly, we analyse the contribution of each proposed VQ-VAE loss component in an additive manner by using the UKB dataset. Afterwards, we look at the features that our Transformer has and how they improve the generative modelling on the ADNI dataset. Lastly, we analyse the effect of model scaling on the overall performance on the UKB dataset. Following [3, 4], we used features extracted by the Med3D network [5] to measure the distribution alignment, via FID and MMD. FID measures the similarity between the synthetic and real datasets by modelling the latent representation of the feature extractor as a multivariate Gaussian and calculating the distance between the two of them via Fréchet distance. On the other hand, MMD measures the distance between the feature means. The Med3D is a 3D segmentation network trained on 23 different tasks, all sharing a common backbone, making it a suitable feature extractor to compute our metrics.

Fig. 1: The influence each loss component has on the perceived quality of the reconstructions. And the losses have compounded representations where for example (2) has both the pixel and frequency components.

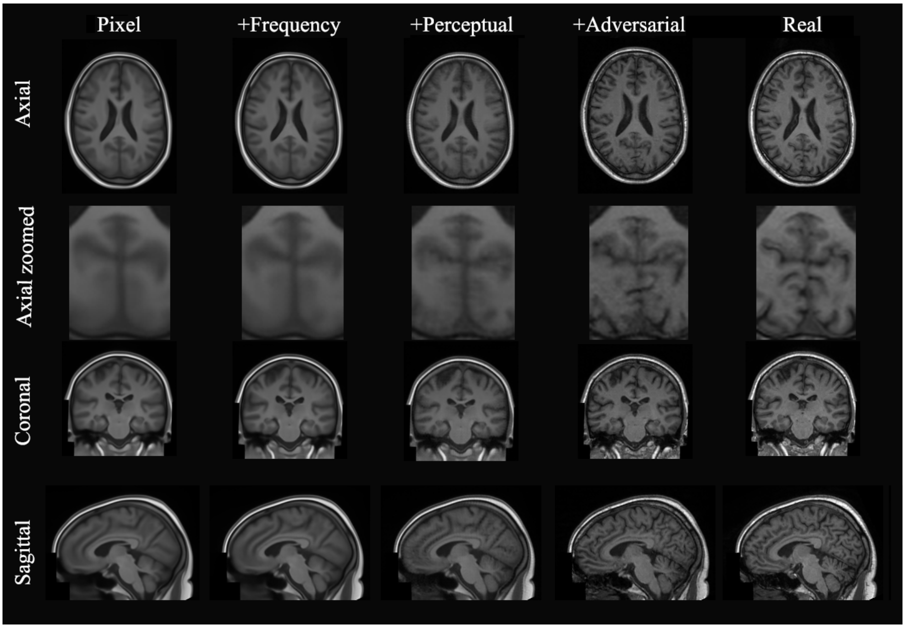

We measured the structural coherence of reconstructions through MS-SSIM and the 4-G-SSIM, where the 4-G-SSIM was shown to be a better surrogate

of a human observer in a wide range of medical imaging situations [6]. This improved characteristic is achieved by processing the images after passing them through a Sobel operator [7] and then weighting the SSIM scores based on a low-level content-based segmentation of the images into homogeneous regions [8].

Table 1: Image fidelity and diversity results of VQ-VAE's loss ablation study

**Loss Component**	**FID** ↓	**MMD** ↓	**MS – SSIM** ↑	**4 – G – SSIM** ↑
Pixel	0.0187	8.344E-06	$0.953_{\pm 0.006}$	$0.782_{\pm 0.018}$
+ Frequency [9]	0.0182	8.136E-06	$0.952_{\pm 0.006}$	$0.779_{\pm 0.018}$
+ Perceptual [10]	0.0147	6.482E-06	$0.952_{\pm 0.006}$	$0.772_{\pm 0.018}$
+ Adversarial [11–13]	0.0004	5.960E-08	$0.949_{\pm 0.006}$	$0.738_{\pm 0.019}$

Supplementary Information Table 1 depicts how each reconstruction loss component influences the four chosen evaluation metrics. The VQ-VAEs were trained for 250 epochs on 70% of the UKB images and were validated on the rest. Each reconstruction loss component has a marginal improvement except for the adversarial loss that improves the FID and MMD by two orders of magnitude, which is showcased in Supplementary Information Figure 1. On the other hand, with better distribution alignment and realism, the structural coherence is marginally reduced due to the limitation of the bottleneck. Each component of the loss that we added to the VQ-VAE has a quantifiable improvement, as seen in Supplementary Information Table 1 and in Supplementary Information Figure 1 where we can see a better definition of the cortical area for perceptual loss and especially for the adversarial one. The downside is that with increased realism of the image comes a reduction in structural preservation, especially in the cortical area as measured by MS-SSIM and 4-G-SSIM. This can be attributed to the informational capacity of the quantization bottleneck of the VQ-VAE.

Table 2: Image fidelity and diversity results of Transformer's ablation study

**Experiment**	**FID** ↓	**MMD** ↓	**MS – SSIM** ↓	**4 – G – SSIM** ↓
Baseline + Live Augmentation	0.0128	1.17E-06	$0.69_{\pm 0.05}$	$0.40_{\pm 0.04}$
+ Root Mean Squared Normalization [14]	0.0150	1.62E-06	$0.71_{\pm 0.06}$	$0.41_{\pm 0.06}$
+ Enhanced Recurrence [15]	0.0081	1.32E-06	$0.66_{\pm 0.08}$	$0.40_{\pm 0.06}$
+ Spatial Relative Positional Bias [16]	0.0077	1.02E-06	$0.66_{\pm 0.08}$	$0.39_{\pm 0.06}$
+ Attention Gating [17]	0.0065	7.45E-07	$0.64_{\pm 0.08}$	$0.38_{\pm 0.05}$
+ Augmentation Conditioning [18]	0.0064	4.76E-07	$0.65_{\pm 0.08}$	$0.38_{\pm 0.09}$

For the Transformer ablation study in Supplementary Information Table 2, we have used the ADNI dataset similarly split into 70% training data and 30% validation. Here, the MS-SSIM and 4-G-SSIM were used to measure the perceptual diversity of the synthetic samples by measuring the mean score of pairs of synthetic images. This works since having lower MS-SSIM/4-G-SSIM indicates lower similarity between the synthetic samples and when looking at a dataset level it means increased diversity. All used features contributed to a

better distribution alignment and diversity, as seen from the FID and MMD, and MS-SSIM and 4-G-SSIM, respectively. In the analysis of the Transformer features, nearly all have a noticeable improvement, as outlined in Supplementary Information Table 2. The only feature that degrades the performance is the Root Mean Squared Normalization.

The most considerable improvement came from the Enhanced Recurrence [15] which allowed our model to have full sequence context at the inference stage, alleviating the exposure bias issue of the teacher forcing training regime [19]. The Spatial Relative Positional Bias [16] brought an across-the-board improvement and, furthermore, we observed during the development that it better modelled the morphological features of the synthetic samples, reducing the prevalence of the morphological features from the distribution tail's which we visually assessed to be abnormally prevalent without it. Even if the Root Mean Squared Normalization [14] degraded the initial performance it substantially improved the convergence as per [20, 21] and synergy with downstream features. Given the existence of the augmentations in the pipeline conditioning, the model on them as per [18] was a natural improvement. This lead to the ability to generate augmentation-free synthetic samples which is a beneficial feature in the medical domain due to reduced post-processing needs.

Table 3: Image fidelity and diversity results of the scale ablation study

**Model**	**#Parameters**	**FID** $\downarrow$	**MMD** $\downarrow$	**MS − SSIM** $\uparrow$	**4 − G − SSIM** $\uparrow$
VQ-VAE Small	6,096,434	0.0005	4.47E-08	$0.926_{\pm 0.007}$	$0.695_{\pm 0.018}$
VQ-VAE Big	39,172,706	0.0004	5.96E-08	$0.949_{\pm 0.006}$	$0.738_{\pm 0.019}$
**Model**	**#Parameters**	**FID** $\downarrow$	**MMD** $\downarrow$	**MS − SSIM** $\downarrow$	**4 − G − SSIM** $\downarrow$
Transformer Small	178,655,537	0.0031	1.17E-06	$0.69_{\pm 0.05}$	$0.41_{\pm 0.02}$
Transformer Big	684,341,553	0.0026	9.53E-07	$0.67_{\pm 0.05}$	$0.40_{\pm 0.02}$

To assess the impact of the model's scale and showcase the need for further scaling up the models that we are presenting in this paper, we trained two versions of the VQ-VAE and two Transformer versions. The difference between the Small and Big models is their capacity; the big models have three times the number of parameters as the small ones. For the VQ-VAE, we reduced the number of filters for the E, D and Dis and the number and size of Q codebook elements. We analysed the scaling behaviour of our pipeline, and it can be seen that with increased capacity, the VQ-VAE and Transformer both model the underlying distribution to a better degree, as shown in Supplementary Information Table 3. The bigger VQ-VAE is at least 20% better when looking at the FID and MMD and, the Transformer is at least 15% better. This, combined with the Train on Synthetic Test on Real results, indicates that there is a potential to significantly increase model performance with further scaling.

C Network Architecture Details

C.1 VQ-VAE

In Supplementary Information Table 4 the parameters for the Small and Big VQ-VAE are outlined and their respective Patch-GAN Discriminators. We also reduced the discriminator's capacity to maintain the balance between the two networks. For overall architecture of the Big VQ-VAE's encoder and decoder are presented in Supplementary Information Table 6 and Supplementary Information Table 7. In Supplementary Information Table 5 the Patch-GAN Discriminator's architecture is outlined.

Table 4: VQ-VAE and Discriminator parameters

**Parameter**	**Big Model Value**	**Small Model Value**
# Residual Layers	3	2
# Channels	256	128
# Embeddings	2048	1028
Embedding Dimension	32	16
Discriminator # Channels	64	32
Discriminator # Layers	3	2

Table 5: Patch-GAN Discriminator

**Layer**	**Filter size, Stride**	**Output size(C, D, H, W)**
Input	-	1 x 160 x 224 x 160
Conv3d	4 x 4 x 4, 2	64 x 80 x 112 x 80
LeakyReLU	-	
Conv3d	4 x 4 x 4, 2	128 x 40 x 56 x 40
BathNorm3d	-	
LeakyReLU	-	
Conv3d	4 x 4 x 4, 2	
BathNorm3d	-	256 x 20 x 28 x 20
LeakyReLU	-	
Conv3d	4 x 4 x 4, 1	
BathNorm3d	-	512 x 19 x 27 x 19
LeakyReLU	-	
Conv3d	4 x 4 x 4, 1	1 x 18 x 26 x 18

Table 6: Encoder part of the Big VQ-VAE

**Layer**	**Filter size, Stride**	**Output size(C, D, H, W)**
Input	-	1 x 160 x 224 x160
Conv3d<br>Relu	4 x 4 x 4, 2<br>-	128 x 80 x 112 x 80
Conv3d<br>ReLU<br>+ Dropout3d	3 x 3 x 3, 1<br>-	128 x 80 x 112 x 80
Conv3d	1 x 1 x 1, 1	128 x 80 x 112 x 80
Conv3d<br>ReLU<br>+ Dropout3d	3 x 3 x 3, 1<br>-	128 x 80 x 112 x 80
Conv3d	1 x 1 x 1, 1	128 x 80 x 112 x 80
Conv3d<br>ReLU<br>+ Dropout3d	3 x 3 x 3, 1<br>-	128 x 80 x 112 x 80
Conv3d	1 x 1 x 1, 1	128 x 80 x 112 x 80
Conv3d<br>Relu	4 x 4 x 4, 2<br>-	128 x 40 x 56 x 40
Conv3d<br>ReLU<br>+ Dropout3d	3 x 3 x 3, 1<br>-	128 x 40 x 56 x 40
Conv3d	1 x 1 x 1, 1	128 x 40 x 56 x 40
Conv3d<br>ReLU<br>+ Dropout3d	3 x 3 x 3, 1<br>-	128 x 40 x 56 x 40
Conv3d	1 x 1 x 1, 1	128 x 40 x 56 x 40
Conv3d<br>ReLU<br>+ Dropout3d	3 x 3 x 3, 1<br>-	128 x 40 x 56 x 40
Conv3d	1 x 1 x 1, 1	128 x 40 x 56 x 40
Conv3d<br>Relu	4 x 4 x 4, 2<br>-	128 x 20 x 28 x 20
Conv3d<br>ReLU<br>+ Dropout3d	3 x 3 x 3, 1<br>-	128 x 20 x 28 x 20
Conv3d	1 x 1 x 1, 1	128 x 20 x 28 x 20
Conv3d<br>ReLU<br>+ Dropout3d	3 x 3 x 3, 1<br>-	128 x 20 x 28 x 20
Conv3d	1 x 1 x 1, 1	128 x 20 x 28 x 20
Conv3d<br>ReLU<br>+ Dropout3d	3 x 3 x 3, 1<br>-	128 x 20 x 28 x 20
Conv3d	1 x 1 x 1, 1	128 x 20 x 28 x 20
Conv3d<br>Relu	4 x 4 x 4, 2<br>-	128 x 20 x 28 x 20
Conv3d<br>ReLU<br>+ Dropout3d	3 x 3 x 3, 1<br>-	128 x 20 x 28 x 20
Conv3d	1 x 1 x 1, 1	128 x 20 x 28 x 20
Conv3d<br>ReLU<br>+ Dropout3d	3 x 3 x 3, 1<br>-	128 x 20 x 28 x 20
Conv3d	1 x 1 x 1, 1	128 x 20 x 28 x 20
Conv3d<br>ReLU<br>+ Dropout3d	3 x 3 x 3, 1<br>-	128 x 20 x 28 x 20
Conv3d	1 x 1 x 1, 1	128 x 20 x 28 x 20
Conv3d<br>Relu	4 x 4 x 4, 2<br>-	256 x 10 x 14 x 10
Conv3d<br>ReLU<br>+ Dropout3d	3 x 3 x 3, 1<br>-	256 x 10 x 14 x 10
Conv3d	1 x 1 x 1, 1	256 x 10 x 14 x 10
Conv3d<br>ReLU<br>+ Dropout3d	3 x 3 x 3, 1<br>-	256 x 10 x 14 x 10
Conv3d	1 x 1 x 1, 1	256 x 10 x 14 x 10
Conv3d<br>ReLU<br>+ Dropout3d	3 x 3 x 3, 1<br>-	256 x 10 x 14 x 10
Conv3d	1 x 1 x 1, 1	256 x 10 x 14 x 10
Conv3d	2 x 2 x 2, 1	32 x 10 x 14 x 10
Quantizer	-	32 x 10 x 14 x 10

Table 7: Decoder part of the Big VQ-VAE

Layer	Filter size, Stride	Output size(C, D, H, W)
Conv3d	3 x 3 x 3, 1	256 x 10 x 14 x 10
Conv3d<br>ReLU<br>+ Dropout3d<br>Conv3d	3 x 3 x 3, 1<br>-<br>3 x 3 x 3, 1	256 x 10 x 14 x 10<br>256 x 10 x 14 x 10
Conv3d<br>ReLU<br>+ Dropout3d<br>Conv3d	3 x 3 x 3, 1<br>-<br>3 x 3 x 3, 1	256 x 10 x 14 x 10<br>256 x 10 x 14 x 10
Conv3d<br>ReLU<br>+ Dropout3d<br>Conv3d	3 x 3 x 3, 1<br>-<br>3 x 3 x 3, 1	256 x 10 x 14 x 10<br>256 x 10 x 14 x 10
ConvTranspose3d<br>ReLU	4 x 4 x 4, 2<br>-	128 x 20 x 28 x 20
Conv3d<br>ReLU<br>+ Dropout3d<br>Conv3d	3 x 3 x 3, 1<br>-<br>3 x 3 x 3, 1	128 x 20 x 28 x 20<br>128 x 20 x 28 x 20
Conv3d<br>ReLU<br>+ Dropout3d<br>Conv3d	3 x 3 x 3, 1<br>-<br>3 x 3 x 3, 1	128 x 20 x 28 x 20<br>128 x 20 x 28 x 20
Conv3d<br>ReLU<br>+ Dropout3d<br>Conv3d	3 x 3 x 3, 1<br>-<br>3 x 3 x 3, 1	128 x 20 x 28 x 20<br>128 x 20 x 28 x 20
ConvTranspose3d<br>ReLU	4 x 4 x 4, 2<br>-	128 x 40 x 56 x 40
Conv3d<br>ReLU<br>+ Dropout3d<br>Conv3d	3 x 3 x 3, 1<br>-<br>3 x 3 x 3, 1	128 x 40 x 56 x 40<br>128 x 40 x 56 x 40
Conv3d<br>ReLU<br>+ Dropout3d<br>Conv3d	3 x 3 x 3, 1<br>-<br>3 x 3 x 3, 1	128 x 40 x 56 x 40<br>128 x 40 x 56 x 40
Conv3d<br>ReLU<br>+ Dropout3d<br>Conv3d	3 x 3 x 3, 1<br>-<br>3 x 3 x 3, 1	128 x 40 x 56 x 40<br>128 x 40 x 56 x 40
ConvTranspose3d<br>ReLU	4 x 4 x 4, 2<br>-	128 x 80 x 112 x 80
Conv3d<br>ReLU<br>+ Dropout3d<br>Conv3d	3 x 3 x 3, 1<br>-<br>3 x 3 x 3, 1	128 x 80 x 112 x 80<br>128 x 80 x 112 x 80
Conv3d<br>ReLU<br>+ Dropout3d<br>Conv3d	3 x 3 x 3, 1<br>-<br>3 x 3 x 3, 1	128 x 80 x 112 x 80<br>128 x 80 x 112 x 80
Conv3d<br>ReLU<br>+ Dropout3d<br>Conv3d	3 x 3 x 3, 1<br>-<br>3 x 3 x 3, 1	128 x 80 x 112 x 80<br>128 x 80 x 112 x 80
ConvTranspose3d	4 x 4 x 4, 2	1 x 160 x 114 x 160

C.2 Transformers

For the Transformer, we base our implementation on the X-Transformer[1] library. In Supplementary Information Table 8 we included the parameters that we used for the X-Transformer default parameters. For the relative positional bias, we did not quantize the difference as in the original work [22] but instead used the full resolution of values.

Table 8: Transformers constructor arguments values

Parameter	Big Model Value	Small Model Value
vocab_size	2048	2048
n_embd	1024	512
n_layers	36	24
n_heads	16	16
emb_dropout	0.001	0.1
ff_dropout	0.001	0.1
attn_dropout	0.001	0.1
position_emb	rotary	rotary
conditioning_type	cross_attend	cross_attend
use_rmsnorm	True	True
attn_gate_values	True	True
shift_mem_down	1	1

[1] https://github.com/lucidrains/x-transformers

D Voxel Based Morphometry Details

Listing 1: Statistical Parametric Mapping 12 segmentation script.

```
clear;
n_cores = 10;
samples = {
    SAMPLES
};

delete(gcp('nocreate'))

parfor (idx=1:size(samples,1),parpool('local',n_cores))
    matlabbatch={};

    %% Adding SPM to path
    addpath('/usr/local/MATLAB/spm12')

    %% Files to be processed
    matlabbatch{1}.spm.spatial.preproc.channel.vols = cellstr(
      {strcat(samples{idx},',1')}
    );
    %% Setting up the segmentations parameters
    spm('defaults', 'FMRI');
    spm_jobman('initcfg');

    matlabbatch{1}.spm.spatial.preproc.channel.biasreg = 0.001;
    matlabbatch{1}.spm.spatial.preproc.channel.biasfwhm = 60;
    matlabbatch{1}.spm.spatial.preproc.channel.write = [0 0];
    matlabbatch{1}.spm.spatial.preproc.tissue(1).tpm = {
      '/usr/local/MATLAB/spm12/tpm/TPM.nii,1'
    };
    matlabbatch{1}.spm.spatial.preproc.tissue(1).ngaus = 1;
    matlabbatch{1}.spm.spatial.preproc.tissue(1).native = [1 0];
    matlabbatch{1}.spm.spatial.preproc.tissue(1).warped = [1 1];
    matlabbatch{1}.spm.spatial.preproc.tissue(2).tpm = {
      '/usr/local/MATLAB/spm12/tpm/TPM.nii,2'
    };
    matlabbatch{1}.spm.spatial.preproc.tissue(2).ngaus = 1;
    matlabbatch{1}.spm.spatial.preproc.tissue(2).native = [1 0];
    matlabbatch{1}.spm.spatial.preproc.tissue(2).warped = [1 1];
    matlabbatch{1}.spm.spatial.preproc.tissue(3).tpm = {
      '/usr/local/MATLAB/spm12/tpm/TPM.nii,3'
    };
    matlabbatch{1}.spm.spatial.preproc.tissue(3).ngaus = 2;
    matlabbatch{1}.spm.spatial.preproc.tissue(3).native = [0 0];
    matlabbatch{1}.spm.spatial.preproc.tissue(3).warped = [1 1];
    matlabbatch{1}.spm.spatial.preproc.tissue(4).tpm = {
      '/usr/local/MATLAB/spm12/tpm/TPM.nii,4'
    };
    matlabbatch{1}.spm.spatial.preproc.tissue(4).ngaus = 3;
    matlabbatch{1}.spm.spatial.preproc.tissue(4).native = [0 0];
    matlabbatch{1}.spm.spatial.preproc.tissue(4).warped = [0 0];
    matlabbatch{1}.spm.spatial.preproc.tissue(5).tpm = {
      '/usr/local/MATLAB/spm12/tpm/TPM.nii,5'
    };
    matlabbatch{1}.spm.spatial.preproc.tissue(5).ngaus = 4;
    matlabbatch{1}.spm.spatial.preproc.tissue(5).native = [0 0];
    matlabbatch{1}.spm.spatial.preproc.tissue(5).warped = [0 0];
    matlabbatch{1}.spm.spatial.preproc.tissue(6).tpm = {
      '/usr/local/MATLAB/spm12/tpm/TPM.nii,6'
    };
    matlabbatch{1}.spm.spatial.preproc.tissue(6).ngaus = 2;
    matlabbatch{1}.spm.spatial.preproc.tissue(6).native = [0 0];
    matlabbatch{1}.spm.spatial.preproc.tissue(6).warped = [0 0];
    matlabbatch{1}.spm.spatial.preproc.warp.mrf = 1;
    matlabbatch{1}.spm.spatial.preproc.warp.cleanup = 1;
```

```
    matlabbatch{1}.spm.spatial.preproc.warp.reg = [
      0 0.001 0.5 0.05 0.2
    ];
    matlabbatch{1}.spm.spatial.preproc.warp.affreg = 'mni';
    matlabbatch{1}.spm.spatial.preproc.warp.fwhm = 0;
    matlabbatch{1}.spm.spatial.preproc.warp.samp = 3;
    matlabbatch{1}.spm.spatial.preproc.warp.write = [0 0];


    %% Starting the job
    spm_jobman('run', matlabbatch);
end
```

Listing 2: Statistical Parametric Mapping 12 smoothing script.

```
clear;
n_cores = 10;

samples = {
    SAMPLES
};

delete(gcp('nocreate'))

parfor (idx=1:size(samples,1),parpool('local',n_cores))
    matlabbatch={};

    %% Adding SPM to path
    addpath('/usr/local/MATLAB/spm12')

    %% Files to be processed
    matlabbatch{1}.spm.spatial.smooth.data = cellstr(
      {strcat(samples{idx},',1')}
    );

    %% Setting up the segmentations parameters
    spm('defaults', 'FMRI');
    spm_jobman('initcfg');

    matlabbatch{1}.spm.spatial.smooth.fwhm = [10 10 10];
    matlabbatch{1}.spm.spatial.smooth.dtype = 0;
    matlabbatch{1}.spm.spatial.smooth.im = 0;
    matlabbatch{1}.spm.spatial.smooth.prefix = '10s';


    %% Starting the job
    spm_jobman('run', matlabbatch);
end
```

Listing 3: Voxel Based Morphometry template script for Cognitively Normal vs Alzheimer's Diseased. The same template was used for Small Ventricles vs Big Ventricles.

```
clear;
%%
addpath('/usr/local/MATLAB/spm12');
%%
spm('defaults', 'FMRI');
spm_jobman('initcfg');
%%
matlabbatch{1}.spm.stats.factorial_design.dir = {
PATHS
};
%%
matlabbatch{1}.spm.stats.factorial_design.des.t2.scans1 = {
CN_SUBJECTS
};
matlabbatch{1}.spm.stats.factorial_design.des.t2.scans2 = {
AD_SUBJECTS
};
%%
matlabbatch{1}.spm.stats.factorial_design.des.t2.dept = 0;
matlabbatch{1}.spm.stats.factorial_design.des.t2.variance = 1;
matlabbatch{1}.spm.stats.factorial_design.des.t2.gmsca = 0;
matlabbatch{1}.spm.stats.factorial_design.des.t2.ancova = 0;
%%
matlabbatch{1}.spm.stats.factorial_design.cov(1).c = [
SEXES
];
%%
matlabbatch{1}.spm.stats.factorial_design.cov(1).cname = 'Sex';
matlabbatch{1}.spm.stats.factorial_design.cov(1).iCFI = 1;
matlabbatch{1}.spm.stats.factorial_design.cov(1).iCC = 1;
%%
matlabbatch{1}.spm.stats.factorial_design.cov(2).c = [
AGES
];
%%
matlabbatch{1}.spm.stats.factorial_design.cov(2).cname = 'Age';
matlabbatch{1}.spm.stats.factorial_design.cov(2).iCFI = 1;
matlabbatch{1}.spm.stats.factorial_design.cov(2).iCC = 1;
%%
matlabbatch{1}.spm.stats.factorial_design.multi_cov = struct(
  'files', {},
  'iCFI', {},
  'iCC', {}
);
matlabbatch{1}.spm.stats.factorial_design.masking.tm.tm_none = 1;
matlabbatch{1}.spm.stats.factorial_design.masking.im = 1;
matlabbatch{1}.spm.stats.factorial_design.masking.em = {''};
%%
spm_jobman('run', matlabbatch);
%%
clear;
%%
spm('defaults', 'FMRI');
spm_jobman('initcfg');
%%
matlabbatch{1}.spm.stats.fmri_est.spmmat = {
PATH_MAT
};
matlabbatch{1}.spm.stats.fmri_est.write_residuals = 0;
matlabbatch{1}.spm.stats.fmri_est.method.Classical = 1;
%%
spm_jobman('run', matlabbatch);
%%
clear;
```

```
%%
spm('defaults', 'FMRI');
spm_jobman('initcfg');
%%
matlabbatch{1}.spm.stats.con.spmmat = {PATH_MAT};
matlabbatch{1}.spm.stats.con.consess{1}.tcon.name = 'Base';
matlabbatch{1}.spm.stats.con.consess{1}.tcon.weights = [1 -1 0 0];
matlabbatch{1}.spm.stats.con.consess{1}.tcon.sessrep = 'none';
matlabbatch{1}.spm.stats.con.delete = 0;
%%
spm_jobman('run', matlabbatch);
```

E Brain Age Model Details

In this study, we used a Simple, Fully Convolutional Network (SFCN) [23] to predict the brainage using the brain images. We were interested in showing the usefulness of the generated synthetic images brain training these models on synthetic data and then evaluating the performance on real ones. Our SFCN follows the same structure as the original one, with the first five blocks containing a 3D convolutional layer (with kernel size = 3), a batch normalisation layer, a max pooling layer and a ReLU activation layer. The sixth block contains a convolutional layer with a kernel size of 1, followed by a batch normalisation layer and a ReLU activation layer. And finally, the seventh block has an average pooling layer, a dropout layer, a fully connected layer and a softmax output layer. The number of channels used in each block was 32, 64, 128, 256, 256, 64, and 40, with the output layer containing 40 classes (representing the age bins).

In our experiments, we performed cross-validation with five iterations to evaluate the model. In training, we used a Stochastic Gradient Descent optimizer with an initial learning rate of 0.1 and a scheduler from the type "step", which multiplied the learning rate value by 0.3 every 97 epochs. We trained the model for 400 epochs where we used weight decay with a coefficient equal to 0.001 for regularization. Finally, for data augmentation, we used the transformations of random Affine (performing rotation, scaling and translation transformations), random variations in intensity levels and contrast, and the addition of random noise.

References

[1] Brudfors, M., Balbastre, Y., Nachev, P., Ashburner, J.: Mri super-resolution using multi-channel total variation. In: Annual Conference on Medical Image Understanding and Analysis, pp. 217–228 (2018). Springer

[2] Brudfors, M., Balbastre, Y., Nachev, P., Ashburner, J.: A tool for super-resolving multimodal clinical mri. arXiv preprint arXiv:1909.01140 (2019)

[3] Sun, L., Chen, J., Xu, Y., Gong, M., Yu, K., Batmanghelich, K.: Hierarchical amortized gan for 3d high resolution medical image synthesis. IEEE Journal of Biomedical and Health Informatics (2022)

[4] Xing, S., Sinha, H., Hwang, S.J.: Cycle consistent embedding of 3d brains with auto-encoding generative adversarial networks. In: Medical Imaging with Deep Learning (2021)

[5] Chen, S., Ma, K., Zheng, Y.: Med3d: Transfer learning for 3d medical image analysis. arXiv preprint arXiv:1904.00625 (2019)

[6] Renieblas, G.P., Nogués, A.T., González, A.M., León, N.G., Del Castillo, E.G.: Structural similarity index family for image quality assessment in

radiological images. Journal of medical imaging **4**(3), 035501 (2017)

[7] Chen, G.-H., Yang, C.-L., Xie, S.-L.: Gradient-based structural similarity for image quality assessment. In: 2006 International Conference on Image Processing, pp. 2929–2932 (2006). IEEE

[8] Li, C., Bovik, A.C.: Content-partitioned structural similarity index for image quality assessment. Signal Processing: Image Communication **25**(7), 517–526 (2010)

[9] Dhariwal, P., Jun, H., Payne, C., Kim, J.W., Radford, A., Sutskever, I.: Jukebox: A generative model for music. arXiv preprint arXiv:2005.00341 (2020)

[10] Zhang, R., Isola, P., Efros, A.A., Shechtman, E., Wang, O.: The unreasonable effectiveness of deep features as a perceptual metric. In: Proceedings of the IEEE Conference on Computer Vision and Pattern Recognition, pp. 586–595 (2018)

[11] Isola, P., Zhu, J.-Y., Zhou, T., Efros, A.A.: Image-to-image translation with conditional adversarial networks. In: Proceedings of the IEEE Conference on Computer Vision and Pattern Recognition, pp. 1125–1134 (2017)

[12] Mao, X., Li, Q., Xie, H., Lau, R.Y., Wang, Z., Paul Smolley, S.: Least squares generative adversarial networks. In: Proceedings of the IEEE International Conference on Computer Vision, pp. 2794–2802 (2017)

[13] Esser, P., Rombach, R., Ommer, B.: Taming transformers for high-resolution image synthesis. In: Proceedings of the IEEE/CVF Conference on Computer Vision and Pattern Recognition, pp. 12873–12883 (2021)

[14] Zhang, B., Sennrich, R.: Root mean square layer normalization. Advances in Neural Information Processing Systems **32** (2019)

[15] Ding, S., Shang, J., Wang, S., Sun, Y., Tian, H., Wu, H., Wang, H.: Ernie-doc: A retrospective long-document modeling transformer. arXiv preprint arXiv:2012.15688 (2020)

[16] Wu, K., Peng, H., Chen, M., Fu, J., Chao, H.: Rethinking and improving relative position encoding for vision transformer. In: Proceedings of the IEEE/CVF International Conference on Computer Vision, pp. 10033–10041 (2021)

[17] Jumper, J., Evans, R., Pritzel, A., Green, T., Figurnov, M., Ronneberger, O., Tunyasuvunakool, K., Bates, R., Žídek, A., Potapenko, A., Bridgland, A., Meyer, C., Kohl, S.A.A., Ballard, A.J., Cowie, A., Romera-Paredes,

B., Nikolov, S., Jain, R., Adler, J., Back, T., Petersen, S., Reiman, D., Clancy, E., Zielinski, M., Steinegger, M., Pacholska, M., Berghammer, T., Bodenstein, S., Silver, D., Vinyals, O., Senior, A.W., Kavukcuoglu, K., Kohli, P., Hassabis, D.: Highly accurate protein structure prediction with AlphaFold. Nature (2021)

[18] Jun, H., Child, R., Chen, M., Schulman, J., Ramesh, A., Radford, A., Sutskever, I.: Distribution augmentation for generative modeling. In: International Conference on Machine Learning, pp. 5006–5019 (2020). PMLR

[19] Arora, K., El Asri, L., Bahuleyan, H., Cheung, J.: Why exposure bias matters: An imitation learning perspective of error accumulation in language generation. In: Findings of the Association for Computational Linguistics: ACL 2022, pp. 700–710. Association for Computational Linguistics, Dublin, Ireland (2022)

[20] Borgeaud, S., Mensch, A., Hoffmann, J., Cai, T., Rutherford, E., Millican, K., Van Den Driessche, G.B., Lespiau, J.-B., Damoc, B., Clark, A., *et al.*: Improving language models by retrieving from trillions of tokens. In: International Conference on Machine Learning, pp. 2206–2240 (2022). PMLR

[21] Rae, J.W., Borgeaud, S., Cai, T., Millican, K., Hoffmann, J., Song, F., Aslanides, J., Henderson, S., Ring, R., Young, S., et al.: Scaling language models: Methods, analysis & insights from training gopher. arXiv preprint arXiv:2112.11446 (2021)

[22] Wu, K., Peng, H., Chen, M., Fu, J., Chao, H.: Rethinking and improving relative position encoding for vision transformer. In: Proceedings of the IEEE/CVF International Conference on Computer Vision, pp. 10033–10041 (2021)

[23] Peng, H., Gong, W., Beckmann, C.F., Vedaldi, A., Smith, S.M.: Accurate brain age prediction with lightweight deep neural networks. Medical image analysis **68**, 101871 (2021)
